# The importance of atrial anatomy

**Published:** 2016-10-03

**Authors:** A. Carlos

Dear Editor,

I have read with great interest the case report published in this Journal by Sabzi,^1^ who described an inadvertent connection between the inferior vena cava (IVC) and the left atrium at the time of the surgical correction of an atrial septal defect (ASD) lying low close to the IVC. The patient was a young 18-year-old male, who required reoperation 24 hours after the initial procedure for re-patching. From a cardiac point of view, the new patch was appropriately anchored and the patient had a good recovery with satisfactory follow-up at 1 year. He required laparotomy for duodenal ulcer perforation.

This case illustrates well the eventual complications at the time of the correction of a sinus venosus ASD of both superior vena cava (SVC) and IVC types with or without the involvement of abnormally draining pulmonary veins. This issue with anatomy was highlighted in an already old contribution by our prior group^2^ and more recently at the time of a review of the technique for the correction of the SVC type.^3^^, ^^4^ Although conceptually simple, the pathology of the atrial septum may become complicated as long as the anatomy, as discussed by Sabzi,^1^ is not appropriately identified.

In this regard, the iatrogenic connection of the major veins, the IVC and SVC to the left atrium, is an almost exceptional complication in current times and this brief report contributes to the understanding of some specific and unfortunate cases. We had the opportunity to treat a similar case of the iatrogenic connection of a major intrathoracic vein, in this case the SVC, after the correction of an SVC, over 30 years ago. An additional coronary fistula was treated, and patching with the redirection of the SVC was performed. This case was diagnosed some years after the correction of a defect. The wrong connections of the IVC might need immediate correction as described in this current report by Sabzi^1^ considering the vast amount of the venous return that depends on the IVC and the immediate impact on the liver function due to massive acute hepatic congestion in addition to cyanosis.

The iatrogenic case reported^1^ might benefit from a slightly more accurate description of the preoperative echocardiogram; nevertheless, this case, as stated earlier, underscores the importance of a meticulous investigation of the pathology of the interatrial septum, although it is usually deemed conceptually and practically easy.
